# State budget transfers to Health Insurance to expand coverage to people outside formal sector work in Latin America

**DOI:** 10.1186/s12913-017-2004-y

**Published:** 2017-02-16

**Authors:** Inke Mathauer, Thorsten Behrendt

**Affiliations:** 1 Department of Health Systems Governance and Financing, World Health Organization, Avenue Appia, 1211 Geneva, Switzerland; 2 Deutsche Gesellschaft für Internationale Zusammenarbeit (GIZ), Friedrich-Ebert-Allee 36, 53113 Bonn, Germany

**Keywords:** Universal health coverage, Vulnerable population groups, Government subsidization of health insurance, Financial protection

## Abstract

**Background:**

Contributory social health insurance for formal sector employees only has proven challenging for moving towards universal health coverage (UHC). This is because the informally employed and the poor usually remain excluded. One way to expand UHC is to fully or partially subsidize health insurance contributions for excluded population groups through government budget transfers. This paper analyses the institutional design features of such government subsidization arrangements in Latin America and assesses their performance with respect to UHC progress. The aim is to identify UHC conducive institutional design features of such arrangements.

**Methods:**

A literature search provided the information to analyse institutional design features, with a focus on the following aspects: eligibility/enrolment rules, financing and pooling arrangements, and purchasing and benefit package design. Based on secondary data analysis, UHC progress is assessed in terms of improved population coverage, financial protection and access to needed health care services.

**Results:**

Such government subsidization arrangements currently exist in eight countries of Latin America (Bolivia, Chile, Colombia, Costa Rica, Dominican Republic, Mexico, Peru, Uruguay). Institutional design features and UHC related performance vary significantly. Notably, countries with a universalist approach or indirect targeting have higher population coverage rates. Separate pools for the subsidized maintain inequitable access. The relatively large scopes of the benefit packages had a positive impact on financial protection and access to care.

**Discussion and Conclusion:**

In the long term, merging different schemes into one integrated health financing system without opt-out options for the better-off is desirable, while equally expanding eligibility to cover those so far excluded. In the short and medium term, the harmonization of benefit packages could be a priority. UHC progress also depends on substantial supply side investments to ensure the availability of quality services, particularly in rural areas. Future research should generate more evidence on the implementation process and impact of subsidization arrangements on UHC progress.

## Background

Latin America has a long social health insurance (SHI) tradition. This had resulted in segmented systems, in which a SHI scheme covers almost exclusively formal sector employees only, and private health insurances those who can afford it. The “rest” of the population, i.e. those in the informal sector, have thereby usually resorted to government health services provided by the Ministry of Health (MOH), resulting in segmented service delivery structures as well [1]. Progressing towards UHC has proven to be difficult in many of the countries with a SHI system, particularly as to the inclusion of population groups outside the formal sector [1–4]. Various vulnerable population groups cannot afford to pay contributions on their own, because they have no income, a very low income, or a very unsteady income.

In fact, there is an emerging consensus among countries on the need to reform health financing systems in order to accelerate progress towards Universal Health Coverage (UHC). UHC means that everyone can access quality health services without facing financial hardship as a result [5]. The 2014 Pan American Health Organization (PAHO) strategy for universal access to health and UHC acknowledges that there are many different ways to progress towards UHC and that each country will need to establish its own action plan, taking into account its social, economic, political, legal, historical and cultural context as well as its priorities and current and future health challenges [6]. The health financing reform steps taken by countries in Latin America during the last decades toward these objectives reflect this diversity [7].

While not the only UHC extension approach, a trend can be seen in Latin America however, in that several countries have moved towards UHC and expanded coverage to people outside the formal sector by transforming the SHI logic that is based on entitlement against mandatory contributions for formal sector employees. These countries have done so by subsidizing health insurance type arrangements through state budget transfers, which serves to cover non-contributing individuals outside the formal sector. This allows to delink entitlements from contributions [8]. Here, a health insurance type scheme is understood as a health financing scheme that usually starts from an insurance logic: it provides coverage of an explicit benefit package to defined and entitled individuals that are identified and affiliated, in exchange for explicit or implicit contributions paid by or on behalf of these. Notably, this type of health financing mechanism and expansion strategy towards UHC is found in countries with strongly varying economic and fiscal situations.

The existing body of literature provides country focused health system reform studies. Specifically, the Lancet 2014 paper series on UHC in Latin America includes a cross-country overview of health systems reforms aiming at UHC in Latin America [9, 10]. Building upon these, this paper fills a gap in the literature: it gives an overview of such arrangements for Latin America and specifically analyses institutional design features of subsidization of health insurance type schemes and their potential contribution to progress towards UHC, with the aim of deriving policy lessons of what works and what does not. The institutional design of a financing arrangement is understood as policy, legal or regulatory specifications that define this arrangement’s structure and the way it operates. Furthermore, this paper is part of a series of regional studies on Europe (Vilcu/Mathauer 2016 for Eastern-European high-income countries [11] and Mathauer et al. [14] on low- and middle-income countries of the European WHO Region [12]), Asia 2016 (Vilcu et al. [13]) and Africa (Mathauer et al. [14]).

The next section presents the methodology and analytical framework applied. The Results section assesses the institutional design features of the schemes as well as progress towards UHC. The Discussion explores possible effects of key institutional design features on UHC and related challenges, followed by a Conclusion.

## Methods

This study chose to consider those Latin American countries with Romance languages, thus excluding the Dutch or English speaking countries and Caribbean island states, most of which with relatively small populations, because they have a different history. In a first step, all countries in this language group were considered if the Global Health Expenditure Database reported social health insurance (SHI) expenditure (or termed social security expenditure for health in WHO’s Global Health Expenditure Database). The second step consisted in identifying countries with a health insurance type arrangement that covers people outside the formal sector by using state budget transfers. Thirdly, countries without SHI expenditure were screened to see whether there is a country with a government subsidization arrangement, but without SHI expenditure. This last step did not expand the list of countries included in this study. Altogether, these study inclusion criteria rendered eight countries to be assessed here, namely Bolivia, Chile, Colombia, Costa Rica, Dominican Republic, Mexico, Peru and Uruguay. As such, countries with a budget funded population-wide national health care system such as Brazil or Cuba, or countries with social health insurance for formal sector employees, but without subsidized enrolment for people outside formal sector work, like El Salvador were excluded.

The study is based on a comprehensive literature search from 1990 to December 2015. Data bases and search engines used in English included Scopus, Science Direct, PubMed, JSTOR and Google, and Scielo and CLASE for Spanish material. In Google, the first five pages, with 10 results per page, were considered, excluding commercial pages and ads, news press or other irrelevant pages. The search terms used for collecting information on institutional design features included: health system OR budget transfer OR health subsidization OR health subsidy OR health insurance OR health vulnerable AND country name AND/OR scheme name. The data search for information on progress towards UHC was based on the following terms: impact health insurance OR catastrophic health expenditure OR impoverish* health care OR out-of-pocket payment OR financial protection OR access health OR utilization health care OR health insurance coverage OR universal coverage OR impact OR effects AND country AND/OR scheme name. Additionally, data for the progress assessment was collected from the WHO’s Global Health Expenditure Database [15], government and country insurance fund webpages. Thus, the study is based on a literature review and analysis of secondary databases. This search strategy generated 70 sources for country information from peer-reviewed journals, studies and reports from United Nations organisations (primarily World Bank, ILO and WHO) and research institutes as well as from reports and legal provisions published by governmental organisations. Most of the studies found were country health financing analyses to review reform experiences, but no studies with explicit impact evaluation design were found.

For each country, respective titles identified through the search process were reviewed, and if found to be relevant the abstract or executive summary was read. If this suggested that the publication could provide information on the institutional design or UHC related indicators, the full publication was assessed. Since the search generated several publications for each country, this allowed for cross-check and triangulation to gather valid and reliable (largely descriptive) information on both institutional design aspects as well as UHC related progress data. Officially published data (e.g. from government) and data found in peer-reviewed journals were used as the preferred source when incoherences were found across several studies. Where in doubt about data, the respective information was checked with WHO country experts. The analytical framework outlined further below guided the information extraction process from the literature, as well as data compilation and organisation.

To identify plausible contributions and patterns of institutional design features in relation to UHC progress, we plotted the improvements in UHC related progress indicators against the respective institutional design features for the eight countries using the analytical framework explained below. Multiple country sources helped to capture changes over time. Where data points of different years were available, progress towards UHC over time and in relation to changes in institutional design could be assessed, however, it was often difficult to do so due to scarce data availability for most of the indicators. This is a limitation to this study, and in view of the multiple factors affecting progress towards UHC, this analysis is of explorative nature.

The paper’s analytical framework to assess the institutional design features of budget transfer arrangements starts from the three health financing functions described in Kutzin [16] and looks specifically at the following features:

- *Revenue raising*
  - Eligibility and enrolment arrangements
  - Financing arrangements
- *Pooling*
  - Pooling architecture
- *Purchasing*
  - Benefit package design and type of providers covered
  - Cost-sharing arrangements
  - Provider payment methods and purchasing arrangements

The detailed analytical framework, which was developed for these regional studies [11–14] is found in Table 1, which specifies the institutional design aspects and outlines how these potentially relate to progress toward UHC. Progress towards UHC refers to improvements in population coverage (here specifically focusing on reported enrolment rates), in financial protection and in access to care. The design features and progress indicators are defined and explained in more detail in the next section, but just to emphasise here that checking whether the poor and lower income quintiles benefit at least proportionally is important for equitable progress towards UHC. It is also important to note that subsidized enrolment and coverage in such schemes is only one possible and plausible factor among several to explain improvements in population coverage, the level of financial protection and access to care of subsidized beneficiaries. The overall economic and fiscal situation is thereby decisive in expanding fiscal space that can be used for state budget transfers to subsidize health insurance contributions. The following Results section and tables present the most recent information available.

**Table 1:** Analytical Framework – Institutional Design Features for Government Subsidization Arrangements^12^

Institutional design aspect	Related policy choices	Intermediate output indicators	UHC progress indicators
Eligibility and enrolment rules			
Groups eligible for exemption from contributions/subsidization	Definition of vulnerability (e.g. children, unemployed, pregnant women, informal sector workers, poor, near poor)	Share of the eligible among the bottom two income quintiles and other vulnerable groups	
Targeting method	E.g. universal (based on a very broad criterion such as residence or no employment in the formal sector), indirect (based on socio-demographic, socio-economic or geographic characteristics usually correlated with poverty and vulnerability), direct (through a means assessment or proxy means testing); different targeting approaches can be in place at the same time for different groups	Share of the exempted/subsidized within total (insured) population; Share of the exempted/subsidized among those being targeted for exemption/subsidization (targeting effectiveness of the system)	
Enrolment process	Active enrolment by the beneficiary or automatic enrolment by the authorities		Total population coverage (i.e. enrolment in health insurance fund), differentiated along income quintiles
Organization responsible for identification of the exempted non-contributors/the subsidized	E.g., insurance company; central, regional, local government		
Type of affiliation/membership	Mandatory or voluntary		
Financing arrangements			
Degree of subsidization/co-contribution	Full or partial (a co-contribution is required)	Share of the exempted/subsidized within total (insured) population/those being targeted for subsidization (importance of government revenue)	
Type of transfer mechanism	Individual-based (a specific amount is being paid for each exempted individual), or lump-sum (a lump sum transfer for the entire exempted population is made)		
Calculation logic to determine the amount of funds to be transferred	E.g., based on regular contribution levels, minimum or average wages, specific percentage of the government budget, negotiated by the government	Sufficient funding for a comprehensive benefit package Level of cross-subsidization from contributions	Financial protection (incidence of catastrophic* / impoverishing health expenditure), also differentiated along income quintiles and other aspects; Access to services
Source of funding for state budget transfers	E.g. general government revenues, earmarked government revenues, transfers from other health insurance funds or from contributors within the same pool (cross-subsidization), donor funding		
Pooling arrangements			
Type of pool(s) (general)	Single pool, or multiple pools	Degree of fragmentation, Size and composition of pools, Level of cross-subsidization	Equity in access (everybody has same access to services along their needs, indepen-dent of their contributions; Equity in financing (every household contributes accor-ding to their ability to pay); Financial protection
Type of pool (exempted/subsidized)	Exempted/subsidized integrated in the pool with contributors, or separate pool for the exempted/subsidized		
Type of health insurance membership of contributors	Voluntary or mandatory		
Purchasing arrangements and benefit package design			
Range of services covered by the benefit package	E.g. comprehensive, inpatient focus, outpatient focus, pharmaceuticals, dental care, indirect costs (e.g. transportation) Different or same package as that for contributors		Financial protection; Access (utilization rates); Equity in access
Type of providers offering the benefit package	Public, private providers		
Degree of cost-sharing	Cost-sharing mechanisms (e.g., co-insurance, co-payment, deductible) and rates		
Provider payment mechanisms	Type of provider payment and rates Same or different rules and rates of provider payment methods	Efficiency	

## Results

### Country and Scheme Overview

Since the start of the millennium, economic growth, changing public demands for the right to health and an extended process of democratization across the region have created windows of opportunity for health financing reforms [9, 17]. Eight countries have now subsidization arrangements. The Dominican Republic, Peru, Mexico, Bolivia and Uruguay have introduced a subsidization arrangement by providing state budget transfers to health insurance type schemes in the 2000s. In contrast, the subsidization arrangements of Chile, Costa Rica and Colombia had already long been in place by then, since 1973, 1979, and 1993 respectively (see Table 2, Column 2). In the case of Chile, a broader and explicit benefit package, called AUGE (“Universal Access to Explicit Guarantees”) came in place in 2003, expanding service coverage and improving coverage equity. These insurance type arrangements were set up with a purchaser-provider split in all countries other than Bolivia, Mexico and Uruguay. All countries studied belonged to the group of upper middle-income countries at the time of the implementation of the subsidization schemes, with the exception of Bolivia, which is a lower-middle income country. Chile and Uruguay are high income countries since 2012 as per the World Bank classification [18].

**Table 2:** Country and Scheme Overview

Country	Name of subsidization scheme (Year of introduction of the subsidization scheme and policy/law)	Own account workers (% of population) [51]	GGHE as % of THE (1995) [15]	GGHE as % of THE (Year of introduction) [15]	GGHE as % of THE (2012) [15]	Social Security Funds as % of GGHE 2012) [51]
Bolivia	“Mother and Child Universal Insurance” *(Seguro Universal Materno Infantil*, *SUMI)* (2002), in existence already since 1996 as the *Seguro Nacional de Maternidad y Niñez*, *SNMN*; “Health Insurance for older persons” *(Seguro de Salud del Adulto Mayor, SSPAM)* (2006) These two insurance type schemes were merged in December 2013 into a new program “the Integrated Health Service of the Plurinational State” *(Servicio Integral de Salud del Estado Plurinacional), that also covers people with disabilities* [28]	33.2	57	63 (2002)	72	37
Chile	“National Health Fund” *(Fondo Nacional de la Salud*, *FONASA)* (1979) 2003: *Plan de Acceso Universal de Garantías Explícitas (AUGE)* [28] – this meant that an explicit guaranteed benefit package came into being. Subsidization of vulnerable groups also through: - Explicit Primary Health Care Interventions Program - Law of catastrophic insurance	n/a	48	38 (2004)	49	9
Colombia	“Subsidized Scheme” *(Régimen Subsidiado)* (1993: “Mandatory Health Plan” *(Plan Obligatorio de Salud)*	43.3	55	-	75	83
Costa Rica	“Costa Rican Social Security Caisse” *(Caja Costarricense de Seguridad Social, CCSS)* (1973: Ley *n.° 5349, de Traspaso de Hospitales*)	18.6	77	-	73	86
Dominican Republic	“Subsidized Scheme” *(Régimen Subsidiado)* (2001: *Ley General de Salud*)	33.2	22	34 (2001)	67	47
Mexico	*Seguro Popular de Salud* (2004: *Reglamento de la Ley General de Salud en Materia Protección Social en Salud*)	n/a	42	44 (2004)	52	56
Peru	“Integrated Health Insurance” *(Seguro Integral de Salud)* (2009: *Ley Marco de Aseguramiento Universal en Salud*)	33.6	54	57 (2001)	61	35
Uruguay	“National Integrated Health System” *(Sistema Nacional Integrado de Salud)* (2007: Law No. 18,211)	21.1	31	56 (2007)	71	60

*THE* Total health expenditure, *GGHE*, General government health expenditure

### Definition of eligible groups

Eligibility rules reflect a country’s understanding of which population groups are considered ‘vulnerable’ with regard to health care access. Hence, how eligibility is defined will affect the proportion of the population ultimately subsidized, and who will remain uncovered. In the eight studied Latin American countries, a wide range of different vulnerability and eligibility criteria is found to determine who benefits from subsidization via health insurance type schemes (see Table 3, column 2). Poverty and low income are the most frequent criteria. Except for Bolivia and Uruguay, countries classify the population into (income) segments as a basis to determine eligibility for benefiting from subsidized coverage. The poorest are in all countries entitled to full subsidization, with the exception of Bolivia where the poor are not specifically defined as an eligible group. In addition, all countries have defined certain vulnerable population groups to be eligible for subsidization based on socio-demographic or socio-economic characteristics. The most frequently covered groups comprise older persons (Bolivia, Chile, Colombia, Dominican Republic, Uruguay), persons with disability (Bolivia, Chile, Costa Rica, Dominican Republic, Uruguay), (rural) self-employed workers (Costa Rica, Peru, Mexico, Colombia), and the unemployed (Dominican Republic, Peru, Uruguay) (see also Table 3). Due to their political situation and historical past, some countries have applied more context-specific eligibility criteria such as internally displaced persons and demobilized combatants (Colombia) or victims of state terrorism and human rights violations (Uruguay and Chile) (see Table 3, Column 2). In Peru, eligibility has recently been granted to all households located in specifically defined and particular poor and disadvantaged areas.

**Table 3:** Eligibility Rules

Country	Groups eligible for subsidization	Targeting method	Organization responsible for identification	Enrolment process	Type of affiliation of the subsidized
Bolivia	Pregnant women; children (<5 years); Older persons (>60 years) that are not affiliated to a social security scheme; people with disabilities [52]	Indirect targeting [52] Post-identification also possible (at the health facility level when seeking care) [53]	Municipal governments, together with Ministry of Health [53]	Enrolment by the municipality [54]	Mandatory
Chile	FONASA group A: The poor; older persons above 80 years; victims of human rights violations and their family members; and those receiving family allowance (children < 18 years, pregnant women, mentally disabled) [55]	Direct targeting: (Proxy) means testing Indirect targeting for beneficiaries of the family allowance and pension [56]	The local administration offices of the health ministry [57]	Active enrolment by the beneficiary [57]	Mandatory
Colombia	The three lowest income groups (out of 6 income groups) [58] Defined vulnerable groups (abandoned children, abandoned older persons, homeless, indigenous people, rural migrants) [59] Shifting towards a universalist approach [21]	Direct targeting: Means testing [27] Indirect targeting of vulnerable groups [24]	The local health authorities at the municipality level [60] Prioritization: Number of the enrolled depends on the resources available in each municipality [24]	Active enrolment by the beneficiary who needs to fill out the beneficiary identification questionnaire (direct targeting) or otherwise provide proof of his/her vulnerability status (e.g. being listed on a specific census list) [60]	Mandatory [21]
Costa Rica	Poor people; indigenous people; people with disabilities Partially subsidized: low-income independent workers [46]	Direct targeting: (Proxy) means testing when attending services and not yet insured [46]	Primary health care units [46]	Once the collected data is validated (household visits) beneficiaries become automatically enrolled [46]	Mandatory [46]
Dominican Republic	Poor; informal sector workers with income below the minimum wage; Disabled; single mothers; unemployed Older persons who receive a “Solidarity Pension” [61]	Direct targeting: (Proxy) means testing Indirect targeting [61]	Regional offices of the beneficiary identification system. Priority is given to areas with high poverty incidence [62]	Active enrolment by the beneficiary [61]	Mandatory [63]
Mexico	Until 2010: Fully subsidized: Poorest four income deciles; families until the 7^th^ decile with a pregnant woman or child, unless otherwise enrolled. Conditional cash transfer program beneficiaries (Oportunidades) Partially subsidized: Households from the 5^th^ lowest income decile to those in the 10^th^ decile, unless otherwise insured; [36] Meanwhile, in practice, nearly everybody without formal sector insurance is fully subsidized [20]	Direct targeting: Means and proxy means testing Indirect targeting for those in Oportunidades [20]	State Government Authorities for Social Protection in Health The number of families being eligible was set and limited, prior to reaching near to universal population coverage rates [64]	Active enrolment on a per-family basis, combined with condition of health check-up in the enrolment units at hospital and clinic level [36]	Voluntary [20]
Peru	Fully subsidized: the poor and extreme poor [65] Partially subsidized: low-income self-employed and families and workers of micro-enterprises [34]	Direct targeting: Proxy means testing [30]	Local Targeting Units of the Household Targeting System [66]	Active enrolment by beneficiaries [66]	Mandatory [34]
Uruguay	People with disabilities; older persons; unemployed; victims of state terrorism [67] Informal sector workers [68]	Indirect targeting for beneficiaries of economic assistance Direct targeting: Applicants required to present information on their income [69]	The local offices of the Public Health Services Administration	Active enrolment by beneficiaries (verification of receiving economic assistance at the local Public Health Services Administration office) [69]	Voluntary [68]

### Targeting and enrolment of eligible persons

In order to identify individuals and households eligible for subsidization all countries use some form of targeting (see Table 3). Most countries studied use both direct and indirect targeting in a complementary way to identify eligible individuals. Direct targeting through (proxy) means testing by determining household income and/or assets are applied in six countries. Households are identified on the basis of surveys (in Chile, Colombia, Mexico, Peru) or via household visits (Costa Rica and Dominican Republic). Three countries (Colombia, the Dominican Republic, Peru) use social assistance identification systems that were originally set up for other social programmes to target individuals for subsidized health insurance. Indirect targeting is applied to include persons on the basis of socio-demographic (e.g., age, sex), socio-economic (occupation, employment status) or socio-geographic characteristics. These characteristics are easily observable and identifiable as well as correlated with vulnerability [19]. Another form of indirect targeting is to include beneficiaries of social assistance programs, namely beneficiaries of a conditional cash transfer program (Mexico), family allowances (Chile and Colombia), pension assistance schemes (Chile, Colombia and Dominican Republic), or social programs for demobilized combatants and internally displaced persons (Colombia).

Since 2010, Mexico has effectively extended eligibility in principle to all those outside the formal sector, abandoning its former direct targeting approach [20, 21]. In Colombia, the central government had equally decided to shift to such a universalist approach, covering all outside the formal sector, however, resource constraints somewhat undermined these intentions [21].

Enrolment is mandatory in all countries except for Mexico and Uruguay. As to the actual enrolment process, both directly and indirectly targeted beneficiaries need to take active steps to get enrolled in all observed countries, except in Bolivia. Getting active implies that the potential beneficiaries need to be well informed about their entitlement to subsidization. In most countries, enrolment takes place at local or regional offices of the government organizations that are in charge of identifying eligible persons (Bolivia, Colombia, Costa Rica, Dominican Republic, Peru, Uruguay), or else at provider level, as in Chile and Mexico. To better reach eligible persons and make enrolment possible when attending health care services, Costa Rica and Peru have established these offices within the local health care facilities. In various cases public media campaigns serve to raise overall awareness for the respective subsidization schemes within the population [22].

### Financing sources

Since the introduction of the subsidization scheme, the general government expenditure on health (GGHE) as a percentage of total health expenditure (THE) has risen in all countries (except Costa Rica where it remained at a high level), the increase ranging from 4 percentage points in Peru to 20% points and 33% points in Colombia (compared to 1995) and the Dominican Republic respectively (see Table 2). The scale of state budget subsidies is not insignificant, as revenue data from other regions suggest [11–14], but precise data is not available for these Latin American countries. Nonetheless, GGHE as a share of THE is still below 60% in two countries (Chile and Mexico).

General government revenues are the main source for financing the subsidization schemes, with the exception of Colombia and Costa Rica (see Table 4, Column 2). Notably, in four countries (Bolivia, Chile, Colombia, Costa Rica), some sources of revenues are ear-marked for state budget transfers, these are predominantly “sin” taxes on tobacco, alcohol or gambling. Chile, on the other hand, earmarks its increased VAT, while Bolivia has an earmarked tax on hydrocarbons. Moreover, in Mexico, Peru, or Bolivia and Colombia, budget transfers come from state, regional or municipal revenues respectively. Notably, only in Peru is the subsidization scheme partly financed by donors, with 6% of its funds coming from donors and regional revenues [23]. Colombia is unique in using cross-subsidies as an important source of revenue for the subsidy scheme: 1.5% of total collections from the contributory regime are transferred to the subsidized scheme [24].

**Table 4:** Financing Arrangements

Country	Financing sources of the subsidization scheme (s)	Calculation logic of subsidy	Type of transfer mechanism	Level of subsidization
Bolivia	General government revenues; District and municipal revenues; Earmarked revenues from a tax on hydrocarbons [70]	Until 2013: For SUMI, 10% of central government’s transfers to the municipalities; for SSPAM, a premium of 56 USD per older person [53] Since 2013 under the Integrated Health Services Program: Budget allocations, and 15.5% of municipal fiscal sources	Lump-sum [53]	Full subsidization
Chile	General government revenues, partly earmarked tax revenues: 1% increase in value added tax, tobacco tax, customs revenues; sale of the state’s minority shares in public health enterprises; Contributions of partially subsidized [71]	Ministry of Finance defines a “Universal Premium” according to available funding and based on inflation-linked currency units [72]	Lump-sum	Full subsidization [27]
Colombia	General government revenue (49% of revenues of subsidized scheme) [73]; Solidarity contributions from the Solidarity and Guarantee Fund (36%) [24]; District and municipal revenues (14%) (including earmarked municipal tax on gambling) [21, 24]	Capitation Payment Unit per subsidized member, prospectively calculated, risk-adjusted based on age (children under 1 year of age, women aged 15–44 and others), sex, and geographic area [74] The average Capitation Payment Unit was US$302 in 2011 (US$506 for those contributing) [35]	Individual-based	Full subsidization
Costa Rica	Earmarked tax revenues on luxury goods, gambling, alcohol and tobacco (80% of subsidy amount) Transfers from the Social Development and Family Assignation Fund (20%), with its funds coming from value added tax and a 5% payroll tax [75]; Contributions of the partially subsidized	Negotiated between the National Social Health Insurance and the Ministry of Finance based on the current minimum wage	Individual-based	Full subsidization; partial subsidization: contributions range from 3.75 to 11.00% of income (depending on the earned amount) [76]
Dominican Republic	General government revenues [32]	n/a	Individual-based	Full subsidization
Mexico	Federal funding: Social contribution (~33% of revenues of Seguro Popular) and the federal solidarity contribution (~49.5%) from general taxes State funding: State solidarity contribution similar in all states (~16.5%) Household contributions (~1%, not implemented) [20]	Three steps: 1) Social contribution is a fixed allocation per enrolled family, amounting to US$70 in 2011 [20] Federal solidarity contribution (FSC): on average 150% of the social contribution (~US$105) [36] State solidarity contribution: Fixed allocation of 50% of the social contribution (~US$35) 2) FSC adjusted based on enrolled individuals, health needs, and performance 3) FSC addresses inequalities among states: if FSC is lower than general federal transfers to states, FSC turns out as an additional budget transfer [20]	Individual-based	Full subsidization [36]: Partial subsidization (although not applied in practice): contributions range from US$60 to US$950 [22]
Peru	General government revenues (over 90% of the subsidized scheme’s revenues); Regional state revenues; International donor funding; Contributions from household for the semi-subsidized (less than 5%) [23]	Ministry of Economy and Finance transfers a pre-determined budget on a historical basis and controls its expansion [30, 34]	Individual-based	Full subsidization; Partial subsidization in the semi-contributive regime: about two thirds of the average expenditure per insured, approxi-mately US$67/year per insured [34]
Uruguay	General government revenues [77]	“Health Quota” per subsidized member, adjusted for sex and age using a formula that reflects evolution of domestic prices, exchange rates and wages [77]	Individual-based	Contributions are fully subsidized

### Level of subsidization

In all schemes, all population groups eligible for subsidization are fully subsidized and do not have to co-contribute, with a few exceptions only. These are largely poor people and the medically vulnerable. The exception applies to low-income informal sector and independent workers in Costa Rica, Peru and Mexico, who are only partially subsidized and who have to co-contribute (see Table 4, Column 5). The contribution rates for self-employed workers in Costa Rica’s semi-contributory regime range from 3.75 to 11.00% of income depending on the earnings. In Mexico, co-contributions for those households above the 5^th^ income decile (outside the formal sector) are supposed to rise with the income, but this rule has not been implemented: Only 1% of the enrolled households had contributed to Seguro Popular in the past, until it moved to a universalist approach. In Peru, the semi-contributory regime applies to about 5% of households, with contributions amounting to about two thirds of the average expenditure per insured.

### Calculation logic of the subsidy

Countries use different approaches to determine the state budget transfer amount for the subsidized (see Table 4, Column 3). The first approach is to define an overall budget as a lump sum transfer, which is negotiated annually and expanded depending on available funding. This approach is used in Chile, Costa Rica and Peru. A second way is to transfer a fixed share of the budget, which in the case of Bolivia is transferred from the national level to the municipalities, where it is pooled with the municipal health budget.

In a third group of countries, the subsidy transfer is based on a per capita amount, which is an estimate of what is needed to purchase the respective benefit package (individual-based calculation logic). This is in place in the Dominican Republic, Colombia and Uruguay. For the latter two, this per capita amount is risk adjusted. Finally, Mexico uses a mixed approach by combining the individual-based calculation logic for the fixed allocation per enrolled individual (“social contribution”) and a risk-adjusted federal “solidarity contribution”. On average, the federal solidarity contribution amounts to 150% of the “social contribution” and depends on the number of enrolled individuals, health needs and the performance of health facilities.

### Pooling architecture

The way resources are pooled determines how the costs of illness are shared across the population. Pooling of resources and health risks is found to take place in two ways: The subsidized are either covered by a separate scheme, or they are integrated and part of a national scheme (see Table 5). The latter option is found in three countries (Costa Rica, Chile and Uruguay), where a single health insurance fund at the national level includes and covers both the subsidized and the contributors, allowing for cross-subsidization. In Costa Rica, 50% of revenues from contributions serve to finance coverage of the non-contributory groups [10]. In Chile, however, this potential for cross-subsidization is reduced to some extent, since usually younger, healthier and high income people decide to opt out from the public health insurance scheme and choose among a number of competing private insurers (Instituciones de Salud Previsional - ISAPREs). In contrast, the other five countries (Bolivia, Dominican Republic, Peru, Mexico, Colombia) have established multiple pools, with a separate scheme for the subsidized and other schemes for formal sector employees. However, cross-subsidization from contributory scheme to the subsidized scheme exists in Colombia, where 1.5% of funds collected from payroll taxes (12.5% of salaries) are transferred to the subsidized scheme. Notably, Colombia is the only country with a full-fledged model of managed competition applying to both the contributors’ and subsidized scheme. The insured can choose from competing insurers/purchasing agencies (Health Promotion Entities for the Subsidized Regime) that are required to ensure the provision of the Mandatory Benefit Package for the Subsidized Regime [25]. This multiple pool system is combined with risk-adjusted per capita allocation mechanism for the purpose of risk equalization across different schemes. Moreover, some countries have set up special funds for “catastrophic” diseases, i.e. illness events that may lead to very high, catastrophic expenditure. Finally, in several countries, fragmentation is further deepened through the existence of separate schemes for various public servant groups, such as the Armed Forces and the National Police [26].

**Table 5:** Pooling Architecture

Country	Single/multiple pool (s) for different population groups	Sub-pools at sub-national levels	Integrated/separate fund for the subsidized	Type of membership of the non-subsidized^a^
Bolivia	Multiple: For the formal sector employees: 9 different social health insurance schemes [78] For the subsidized: SUMI et SSPAM, and since December 2013the “Integrated Health Service of the Plurinational State” Government Program [52, 70]	Sub-pools at the municipal levels [70]	Separate scheme for the subsidized	Mandatory
Chile	Multiple: Fondo Nacional de la Salud, FONASA for 80% of the population [31] 7 private Health Insurance Institutions (*Instituciones de Salud Previsional*, *ISAPREs*) for 16% of the population that opted out [27]	Regional health entity pools that determine the budget for all public health care providers in their area [71]	Integrated: Subsidized, semi-contributive and contributory regimes form FONASA	Mandatory [71]
Colombia	Multiple: Solidarity and Guarantee Fund (*Fondo de Solidaridad y Garantía*, *FOSYGA*) at national level, with two separate accounts: 1) Contributory scheme (*Regimen Contributivo*) 2) Subsidized scheme (*Regimen Subsidiado*) FOSYGA also operates as a cross-subsidization mechanism between the contributory and subsidized scheme 21	Multiple competing purchaser structure with a twofold fragmentation: From national to municipal level and from the municipalities to the purchasers (both risk-adjusted) [79]	Separate scheme, but receives 64% of FOSYGA funds (including 1.5% of revenues from formal sector contributions as cross-subsidization) [21]	Mandatory [80]
Costa Rica	Single [27]	No sub-pools	Integrated into the National Insurance Fund	Mandatory [75]; Voluntary for self-employed [46]
Dominican Republic	Multiple: Contributory scheme Subsidized scheme 32 “Catastrophic illnesses fund” for the subsidized (*Fondo para enfermedades catastróficas*) [81]	No sub-pools	Separate scheme for the subsidized	Mandatory [32]
Mexico	Multiple: “Mexican Social Security Institute” *(Instituto Mexicano del Seguro Social, IMSS*) for the formal sector; “Institute for Social Security and Services for Government employees” (*Instituto de Seguridad y Servicios Sociales de los Trabajadores del Estado*, *ISSSTE*) [27] “Seguro Popular”, which consists of 3 pools - Fund for Allocations of Health Services (*Fondo de Aportaciones para los Servicios de Salud*, *FASSA*), - Fund for Protection against Catastrophic Expenses (*Fondo para la Protección contra Gastos Catastróficos*, *FPGC*), - Medical Insurance for a New Generation (*Seguro Médico para una Nueva Generación*, *SMNG*) [36]	State level sub-pools [36]	Separate scheme for the subsidized	Mandatory
Peru	Multiple: Social Health Insurance (*Seguro Social de Salud*, *EsSalud*) for formal sector workers, Separate funds for the Armed Forces and the National Police respectively, *Seguro Integral de Salud*, *SIS* for the subsidized regime and the semi-contributive regime and “Intangible Solidarity Fund for Health” (*Fondo Intangible Solidario de* Salud, FISSAL) for catastrophic diseases of the subsidized [34]	No sub-pools	Separate scheme for the subsidized	Mandatory for formal sector employees; voluntary for self-employed [34]
Uruguay	Multiple “National Health Fund” (*Fondo Nacional de Salud*, *FONASA*) A separate fund for the Armed Forces (*Sanidad Militar*) and the National Police (*Sanidad Policial*) respectively [68]	No sub-pools	Integrated into FONASA	Mandatory for formal sector workers [68]

^a^This usually includes formal sector employees and pensioners as well as the self-employed paying income tax

Beyond fragmented, separate schemes, fragmentation also occurs along territorial lines. In Mexico, in Chile, and in Colombia and Bolivia, actual fund flows and pooling occurs at the state, regional and municipal level respectively. However, in Colombia, with the highest number of sub-pools (about 1,000), as well as in Mexico, central government funds are allocated on the basis of risk adjustment criteria to the sub-national pools. Mexico uses a federal solidarity contribution with the aim to adjust socio-economic inequalities across states with poorer states receiving more funding. In Colombia, prospective risk adjusted allocations for the sub-pools at the municipal level are based on age, sex and geographic area.

Last not least, the level of risk pooling is also determined by the type of membership. Mandatory affiliation is known to result in a more balanced risk composition and hence stronger redistributive capacity in contrast to voluntary affiliation. It is found that the subsidized are affiliated on a mandatory basis in all countries except for Mexico and Uruguay. However, in the Dominican Republic mandatory affiliation is actually not fully implemented. Moreover, as affiliation of formal sector employees is also mandatory in all countries, this further strengthens risk pooling in those countries with an integrated scheme. Yet, as Chile allows opting out to private health insurance schemes, this results in fragmentation and population groups with higher risks are concentrated in FONASA [27].

### Benefit package design: Scope of services and type of providers covered

Purchasing is defined as the allocation of pooled funds to the health care service providers on behalf of the population for a defined benefit package. The definition of the range of services covered in the benefit package is decisive for the level of financial protection and access to care, as are the cost-sharing mechanisms. In general, over the recent decade, a real expansion of the benefits covered is notable [9, 28]. Uruguay, which has introduced an “Integrated Health Care Plan” without specifically defining its contents, and Costa Rica have broad benefit packages, although rationing occurs through waiting lists for example. The other six countries have chosen to specify the explicitly guaranteed health services covered for all insured or for specific insured groups, e.g. through a health care plan or a list that defines by law which health conditions or clinical procedures are covered or not covered. The scope of these lists, however, varies (Table 6, Column 2). For Chile, Mexico and Colombia (since 2012), this is a relatively comprehensive package, covering in-patient, out-patient and specialized care as well as drugs. Compared thereto, the Dominican Republic and Peru have less comprehensive benefit packages for the subsidized, particularly in terms of in-patient care, specialized or high cost services. Moreover, four countries cover selected high-cost treatments through a separate catastrophic illnesses fund (Dominican Republic, Mexico, Peru, Uruguay). Over recent years, increases in available funding (in Chile, Mexico), but also, among other reasons, judicial trials (in Colombia, Costa Rica, Uruguay) have gradually expanded the range of benefit packages towards more specialized care interventions [29].

**Table 6:** Benefit Package Design and Provider Payment Methods

Country	Range of services covered by the benefit package	Degree of cost-sharing for the subsidized	Degree of portability	Provider-payment mechanisms
Bolivia	SSPAM: outpatient care, diagnostic services, dental care, hospitalisation, drugs SUMI: eventually extended to 547 services related to pregnancy, neonatology, paediatrics, dental care, diagnostic services, and all other services not on the list of excluded services (such as high cost interventions, chemotherapy, radiotherapy, transplantations, orthodontics) [70]	None [53]	National portability [54]	Primary care: fee-for-service Higher levels: Budget allocations [53]
Chile	Plan AUGE started with 53 pathologies of outpatient, inpatient and specialist care services and includes 80 pathologies since 2005 [47, 82] Extension of Plan AUGE has to remain within the established amount of the “Universal Premium” within the next 12 months FONASA also provides coverage of extended primary health care and catastrophic diseases [71]	None for FONASA groups A and B [47]	National portability [31]	Primary care: Fixed rate per capita and a budgeted amount; Public hospitals: 50% historical budget; 50% payment for “treatment package”; Ambulatory hospitals: Pay-per-visit rate (fee-for service) for AUGE services Catastrophic diseases: per case payment Private hospitals: Diagnosis Related Groups [27]
Colombia	Mandatory Benefit Package for the Subsidized Regime: Outpatient care, specialized care for catastrophic illnesses, limited coverage for most inpatient care [21, 79] Since 2012, the benefit packages of the contributory and subsidized scheme are harmonized [83]	Lowest income group: no co-payment; 2^nd^ and 3^rd^ lowest income level: co-insurance of 10% Ceiling: monthly minimum wage [84]	National portability [85]	Preventive and primary care services: Capitation Specialist and hospital care: Fee-for-service [79]
Costa Rica	No explicitly defined benefit package nor positive list: drugs and services at all levels of care are covered [86] Implicit rationing through waiting lists [46]	None [49]	National portability in case of emergencies [87]	Primary Care Units: Capitation, adjusted for sex, age and area-specific infant mortality [88]; Secondary and tertiary care: Budgets; fee for service for medicines) [27]
Dominican Republic	Health promotion and disease prevention, primary health care, in-patient and surgical care services, outpatient care services and drugs, diagnostic tests, preventive dental care, complementary provisions for people with disabilities. The cost of the benefit package of the subsidized regime is of US$60 per year (US$240 per year for the contributive regime) [32] Catastrophic illnesses fund (*Fondo para enfermedades catastróficas*) [81]	None [89]	n/a	Fee-for-service [32]
Mexico	Fund for Allocations of Health Services: Essential in-patient and out-patient care services (the Universal Health Services Catalogue includes 284 interventions and 522 drugs) Fund for Protection against Catastrophic Expenses: Specialized care services Medical Insurance for a New Generation: any other services not covered above for children below 5 years (131 interventions) [20]	None^907^	National portability [91]	Universal Health Services Catalogue interventions: Capitation payment Catastrophic illnesses: Per-case payment [22]
Peru	Essential Health Insurance Plan: 140 health interventions and services (covering about 65% of disease burden) [23, 30, 34]	None [33]	n/a	Fee-for-service [30]
Uruguay	Integrated Health Care Plan for all insured: Broad benefit package with services at all levels of care and drugs [77]	None [92]	n/a	Risk-adjusted per capita payment [68], piloting of Diagnosis Related Groups [93]

The covered benefit package for the subsidized is largely purchased from and delivered through government providers. However, in order to secure that services of the benefit package are actually provided, these can also be obtained from private providers in Colombia [21], Peru [30] and Chile [31]. Nonetheless, access to health services is unequal in that respect, since contributors have a more direct access to private providers, in particularly in the countries with separate schemes [28]. Last but not least, most countries have introduced portability of coverage within the whole country, such that beneficiaries have access to services beyond the area where they are registered.

### Cost-sharing mechanisms and rates

In all the countries studied emergency care and some specific key (primary) health care services offered by public facilities of MOH can be accessed by everyone, regardless of insurance status, but co-payments are required [20, 21, 32, 33]. In contrast, for the covered benefit package the fully subsidized are completely exempt from any form of co-payments in all countries except in Colombia (Table 6, Column 3). In Colombia, the subsidized in the 3^rd^ and 4^th^ income groups (out of four categories and thus not belonging to the poorest), have to make co-payments of 10% up to a ceiling of a monthly’ minimum wage.

### Provider payment methods and purchasing arrangements

Separate purchasing agencies are in place in all countries other than Mexico, Bolivia and Uruguay. But even when there is a separate purchasing agency, the separation of functions remains incomplete in countries where the purchaser channels funds to decentralised government health authorities that pool them again with sub-national budgets, such as in Chile. Beyond institutional design, there are also capacity challenges. For example, the Dominican Republic [32] and Peru [34] are reported to have experienced some problems to effectively contract providers. Potential advantages of a purchaser-provider split can thus not be pursued in its full potential, such as a move towards more strategic purchasing.

Related thereto, the types of provider payment methods in place have implications on provider behaviour and hence on access to care. As Table 6 shows, countries use a diversity of combinations of paying for both outpatient and inpatient care and there are efforts to move towards more strategic purchasing by using provider payment methods that set incentives for more efficient provider behaviour. For example, case payment is used in two and capitation is used in four countries respectively. Fee-for-service, nonetheless, remains very dominant. Most importantly, all countries studied use the same provider payment methods for the subsidized and those in the formal sector schemes, with the exception of Mexico. However, average per capita spending for the subsidized is found to be lower than for the contributors in a number of countries [32, 35].

### Progress on universal health coverage indicators

#### Population coverage

In view of the focus on subsidized health insurance type schemes here, population coverage is understood as the percentage of the population that is enrolled to a health insurance scheme. Total population coverage thereby indicates the comprehensiveness of the health insurance system in a country. It is important to note, though, that reported enrolment rates cannot be equated with effective access to health services. On the one hand, enrolment may not mean that people are already (or again) in possession of a (renewed) insurance card. Moreover, non-availability of services and other supply side constraints such as staff shortages and quality concerns limit access.

As Table 7 shows, total enrollment rates are above 95% in Uruguay, Chile, Colombia, and even higher in Costa Rica and Mexico. It is somewhat lower in Peru (72% in 2012) and the Dominican Republic (69% in 2016). Some of the countries studied have made substantial progress in enrolment rates within relatively short time. In Mexico in the early 2000s almost half of the population was without any type of insurance coverage. Since 2003, more than 41% of the population got insured under the *Seguro Popular* [36]. In Peru, enrolment has increased from 37% in 2004 to 72% in 2012 [33, 34]. The Dominican Republic has almost tripled the number of insured people within nine years; from 27% in 2007 to 55% in 2013 and 69% in 2016 (see Table 7, Column 2). Costa Rica and Colombia started much earlier on this road with impressive results as well. Chile and Uruguay have increased their already high insurance coverage rates from 89% in 2007 to 94% in 2012 and from 86% in 2007 to 95% in 2013 respectively. Hence over the last decade, all countries have made considerable progress in enhancing enrolment rates.

**Table 7:** Insurance Enrolment Rates

Country	Insurance Enrolment Rates of Total Population (in %)	Share of subsidized within total population (in %)	Share of subsidized within total insured population (in %)	Exclusion error	Inclusion error
Bolivia	43% (2008) [94]	12% (2008) [94]	28% (2008) 42% (2012)	n/a	n/a
Chile	89% (2004); 94% (2012) [95]	20% (2012) [95]	19% (2012) [95]	n/a	n/a
Colombia	89% (2008) [79]; 96% (2010) [21]	53% (2014) [79]	58% (2014) [79]	2% (2013) [21]	16% (2013) [21]
Costa Rica	87% (2003); 98% (2014) [27]	11% (2014)	11% (2010) [75] 10.6% (2014)	10% (2010) [46]	n/a
Dominican Republic	40% (2009) [32]; 55% (2013) [96], 69% (2016) [97]	25% (2013) [96]	46.% (2013) [96]	68% (2009) [32]	n/a
Mexico	57% (2003); 98% (2012) [36]	44% (2012) [36]	44% (2012)	10% (2009) [22]	n/a
Peru	64% (2010); 72% (2012) [33]	39% (2012) [33]	54% (2012) [33]	16% (2013) [30]	12% (2003)
Uruguay	86% (2007); 95% (2013) [77]	5% (2013) [77]	5% (2013)	n/a	n/a

Looking specifically at the share of the subsidized as of total insured or total population gives an idea of the magnitude of the subsidization arrangement. In fact, there are large differences across countries (see Table 7, Columns 3 and 4). In Chile, Costa Rica and Uruguay, with coverage rates above 90%, the share of the fully subsidized among the total insured population is only 20%, 11% and 5% respectively, since most of the population are contributors. This share is between 40-45% in Mexico, Bolivia and the Dominican Republic, yet also due to overall lower insurance coverage rates in the latter two countries. Colombia, with a high total insurance coverage rate, has the largest share of subsidized people within their insured populations (58%). In Peru, on the other hand, the share of the subsidized among the insured is equally large (54%), but the total enrolment rate is still lower at 72%. The magnitude of the subsidization scheme is a function of the size of the formal sector, but also of the political commitment to define eligibility for subsidization more broadly.

Moreover, it is important to reveal whether and how effectively the subsidization schemes reach the target beneficiaries. Table 7 (Columns 5 and 6) also presents data related to targeting effectiveness: The inclusion error refers to the share of non-eligible individuals being enrolled in the subsidization scheme. The exclusion error refers to individuals that are in principle eligible but that are not enrolled. The high exclusion errors in Peru and in particular in the Dominican Republic are noteworthy and of concern. This may relate to the primarily direct targeting approach applied in those two countries. However, the exclusion error also needs to be seen in relation to the size of the eligible population. For example, in Costa Rica with 12% being eligible for subsidization, the 10% exclusion error affects 1% of the total population.

With respect to the inclusion error, on the other hand, more recent data is only available for Colombia and Peru, however, wrong inclusion is also reported for other countries. The share of non-eligible persons among those being subsidized had been high in Mexico and ranged from 54% to 60% in the years after the introduction of the *Seguro Popular* [37, 38]. One reason related to the way Seguro Popular is financed. There is no incentive for the State Government Authorities for Social Protection in Health to assess household income to determine household contributions to be collected upon enrolment. Instead, federal transfers to the states depend on the number of enrolled individuals and assessing income and collecting contributions from them would reduce their willingness to enrol [20]. As a result, a high enrolment rate was achieved at the expense of a high inclusion error that implied large amounts of public funds going to households not in need for it [37].

#### Financial protection

Global evidence has shown that there is a strong correlation between out-of-pocket expenditure (OOP) as a share of total health expenditure (THE) and the share of households experiencing financial hardship [39]. Thus, a starting point is to look at trends in OOP as a share of THE. Financial protection, specifically, can be measured in two complementary ways. One is to measure the incidence of catastrophic expenditure. This occurs when a household’s total OOP equal or exceed 40% of the household’s non-subsistence spending, i.e. their capacity to pay, as per the WHO definition [40]. Different (lower) thresholds of determining catastrophic expenditure have also been proposed in the literature and are being used (and reported here). The second measure is impoverishing health expenditure that occurs when OOP pushes a household below the poverty line or even deeper into poverty [39].

Across the Latin American countries studied here, three countries (Peru, Chile, Mexico) have a relatively higher share of OOP expenditure (between 29% and 44% in 2014) compared to the other five countries, where OOP expenditure as of THE ranges between 15% and 25% in 2014. However, other than in Costa Rica and Uruguay with slight increases, OOP has been decreasing [15]. Data in Table 8 (Column 3) also confirms that countries with a higher OOP share tend to have a relatively higher incidence of catastrophic health expenditure. In fact, the lowest incidence of catastrophic health expenditure (at a 40% threshold level) is found in Costa Rica (0.4%), whereas it is considerably higher in Chile (6.4%) and in the Dominican Republic (9.8%, however at a 30% threshold), despite the latter’s moderate OOP level of 21%. Moreover, disaggregated data reveals that a high percentage of households from the lowest two income quintiles faces catastrophic health expenditure at a 25% threshold in Chile and Colombia (15 and 10% respectively), while this percentage is the lowest in Costa Rica (1%).

**Table 8:** Financial Protection and Access to Health Care Services

Country	Increase/decrease of OOP as % THE since scheme introduction [15]	Incidence of catastrophic health expenditure as % of households *(at 40% of households’ capacity to pay, or otherwise indicated)*	Impoverishing health expenditure as a % of population	Changes in utilization rates of health care services after introduction of subsidization scheme
Bolivia	- 6% (1996^a^-2014)	3.75% (2002) [98] 3.3% (2006) [99]	n/a	n/a
Chile	- 10% (2004^b^-2014)	6.4% (2012) [99] At 25% threshold: 21% (2010) 15% of households in the 1^st^ and 2^nd^ income quintiles (2006) [7]	Poverty headcount $2.00: 1.2% (2006) Poverty headcount $1.25: 0.4% (2006) [7]	3% increase in utilization of outpatient health services among the 1^st^ and 2^nd^ income quintiles from 2003 to 2011 [7]
Colombia	- 23% (1995–2014)	Slight improvement (for data from 2005–2010) [100] 2.8% (2012) [99] At 25% threshold: 8% (2010) 13% of households in the 1^st^ and 2^nd^ income quintiles (2008) 10% of households in the 1^st^ and 2^nd^ income quintiles (2010) Catastrophic payments are concentrated among the poor [7]	Slight improvement (for data from 2005–2010) [100] Poverty headcount $2.00: 1.8% (2008); 1.5% (2010) Poverty headcount $1.25: 1.2% (2008); 0.7% (2010) [7]	50% increase in the use of health services among the poorest and the rural population from 1995 to 2005 [24]
Costa Rica	+4% (1995–2014)	0.4% (2012) [99] At 25% threshold: 1% (2010) Below 1% of households in the 1^st^ and 2^nd^ income quintiles (2004) 1% of households in the 1^st^ and 2^nd^ income quintiles (2013) [7]	Poverty headcount $2.00: 0.3% (2004); 0.1% (2013) Poverty headcount $1.25: 0.1% (2004); below 0.1% (2013) [7]	n/a
Dominican Republic	- 24% (2001–2014)	At 30% threshold: 9.8% (2012) [99]	n/a	n/a
Mexico	- 9% (2004–2014) 2002: 58% of total OOP from the uninsured and Seguro Popular enrolees 2010: 36% of total OOP from the uninsured and Seguro Popular enrolees [36]	2.4% (2012) [99] 2000: 3.1% catastrophic health expenditure 2010: 2% catastrophic health expenditure [36] At 25% threshold: 1% (2010) 2% of households in the 1^st^ and 2^nd^ income quintiles (2000) 1% of households in the 1^st^ and 2^nd^ income quintiles (2010) [7]	Poverty headcount $2.00: 0.9% (2000); 0.2% (2010) Poverty headcount $1.25: 0.3% (2000); 0.1% (2010) [7]	In 2010 (compared to 2000) utilization rates of public health care services had increased for those previously uninsured (e.g. proportion of births in Ministry of Health facilities increased from 32% to 48%) [36] Increased access to drugs and to the treatment for chronic diseases [37] 1% decrease in utilization of outpatient health services among the 1^st^ and 2^nd^ income quintiles from 2006 to 2012 [7]
Peru	−7% (2001–2014)	4.0% (2012)^104^ At 25% threshold: 5% (2010) 4% of households in the 1^st^ and 2^nd^ income quintiles (2008) 5% of households in the 1^st^ and 2^nd^ income quintiles (2011) [5] Slight improvement since 2009	Poverty headcount $2.00: 1% (2004); 1.1% (2011) Poverty headcount $1.25: 0.6% (2004); 0.7% (2011) [5]	Increase of 13 percentage points between 2000 and 2004 [74] Proportion of institutional deliveries increased from 24% of births in rural areas and 58% of births in urban areas in 2000 to 58% in rural and 85% in urban areas in 2012 [30]
Uruguay	+3% (2007–2012)	n/a	n/a	n/a

^*a*^Introduction of subsidization for women [100]

^b^Introduction of AUGE

#### Access to health care services

Enrolment rates and financial protection indicators do not reveal whether people actually have effective access to needed health services, i.e. the ability to receive health services they need implying the availability, affordability and acceptability of services, or whether they forego seeking care [41]. This is difficult to measure directly, as need cannot be easily captured for all health services. Utilization rates for inpatient and outpatient care are therefore used as proxy indicators. Comparing utilization rates before and after the introduction of the subsidization scheme and between the insured and uninsured over time, or if that data is not available, across income quintiles, may thereby reveal improvements or prevailing inequities in access to care.

Data on health service utilization rates before and after the introduction of the subsidization schemes were only available for four countries. Although utilization rates remained lower than the ones of contributors, access to health care services has improved for the poorest income quintiles in all these countries (see Table 8, Column 5). This increase has been very high in Colombia, where in comparison to the remaining uninsured, a 50% increase in the use of health services among the subsidized urban poor and the rural population could be observed over the period of 1995 to 2005.

## Discussion

### Effects of eligibility criteria and targeting approach on population coverage

This section explores the plausible contribution of institutional design features relating to eligibility and enrolment rules on UHC progress and also highlights particular challenges. The way eligibility for subsidization is defined together with the specific targeting method seem to be decisive for insurance coverage rates of the subsidized groups.

Eligibility criteria and what is considered as vulnerable vary somewhat across the countries, although most countries pursue several definition logics to capture the most vulnerable population groups, which partly overlap. All countries except Bolivia originally put a strong focus on the poor, identifying them on the basis of assessing their means and income. Another focus is on socio-demographically defined groups (children, older persons, pregnant women or mothers). A third eligibility logic is to include social security or social assistance recipients, e.g., the unemployed, disabled and family allowance beneficiaries, such as in Uruguay, Mexico, Dominican Republic and Chile.

However, there remain critical coverage gaps of some specific economically or medically vulnerable population groups. It is found that illegal immigrants and people without documents are not explicitly mentioned as being eligible for subsidization in all countries studied, and hence they remain excluded [42] as are indigenous people (in Peru for example) [43] and internally displaced persons [44] in some countries and areas. Another group are the officially unemployed [45]. Also, many of Peru’s uninsured are not poor but low-income and would be eligible for the semi-contributive regime. Still, enrolment of these persons is difficult to implement and as in other countries across the globe, partial subsidization has not considerably increased population coverage. Limited resources also determine the definition of eligibility criteria. For example, in Bolivia, due to resource constraints, eligibility had been restricted to pregnant women, children and older persons, until coverage was slightly expanded to include people with disabilities in 2013 under the new scheme.

As to the effect of different targeting approaches, evidence suggests that indirect targeting may be more prone to inclusion errors. Also, it appears that direct targeting is one contributing factor for higher exclusion errors, as exampled by Peru and the Dominican Republic (with a primarily direct targeting approach) as well as Mexico in earlier years. But the exclusion error can also be due to cumbersome enrolment procedures, as found in Costa Rica with a 10% exclusion error relating to 12% of the population being eligible for subsidization [46]. The near to universalist approach that Mexico applies since 2010 in practice results in close to 100% population coverage rates. Yet, this is realized at the cost of an inclusion error of people that should be covered by the formal sector insurance schemes.

Due to limited evidence, it remains unclear to what extent the design feature of mandatory membership enhances enrolment rates and whether active enrolment by the authorities to facilitate enrolment is decisive even under mandatory affiliation. Moreover, the chosen targeting approach does not always coincide with fiscal reality and resource availability. This was the case in Colombia for example, where some municipalities were not able to mobilize sufficient resources for their per capita budget transfer to subsidize the enrolment of all eligible people outside the formal sector [21, 26]. However, generally speaking, if municipal funding is required to enrol eligible individuals, the incentive to enrol may not be as strong. In Colombia, as a result, certain vulnerable groups were prioritized over others [24]. In recent years, however, this challenge has been overcome. Likewise, in the Dominican Republic and Peru, resource constraints and slow implementation of their targeting approaches (also applied in other social assistance/protection programmes) equally explain the high exclusion error thus preventing the actual population coverage potential as per their eligibility rules [30, 32].

### Effects of the pooling architecture on equity in access

Some variation is found among countries with respect to critical pooling design aspects, namely scheme integration versus scheme separation as well as mandatory versus voluntary membership. This determines the size and composition of the risk pool and thus the level of fragmentation as well as the scope for cross-subsidization within the system. Among the three countries with an integrated pool, Costa Rica stands out with a high degree of cross-subsidization from the contributors to non-contributors. It is also notable that for the five countries with separate schemes, benefit packages differ despite harmonization efforts in some cases, and in fact, in these countries, the subsidized have access to a smaller benefit package than the contributors.

Only one of these countries (Colombia) has established a direct cross-subsidy mechanism across different schemes in addition to risk-adjusted per capita allocations for the subsidized scheme. This, however, did not overcome inequalities in access to services and thus perpetuates differences in financial protection of those in the subsidized scheme versus those in the contributory scheme. Hence, the coexistence of multiple schemes in which different socio-economic groups are covered by different funding pools, maintains or even increases inequalities in access and financial protection in the countries with separated schemes.

Mandatory coverage increases risk pooling, even in the four countries with separate schemes. When coupled with an integrated risk pool, solidarity in financing and the scope for cross-subsidization is larger, such as in Uruguay and Costa Rica. With its opting out option, Chile (with an integrated scheme) is the only country with substitutive private health insurance, thus limiting overall solidary and equity in financing and access. Moreover, ISAPREs members can switch back to FONASA, especially above a certain age or when suffering from severe illness, when their premiums and co-payments increase. As such, FONASA is burdened with the high-risk members, whereas ISAPREs engages in risks-selection to enrol low-risk members [47]. Reform discussions are underway on how to reform and unify the two sub-systems of FONASA and ISAPREs.

Countries with sub-pools at sub-national levels are particularly prone to the problem of unequal access to health services when there are different socio-economic conditions and disease burden across sub-national units. Therefore, Colombia and Mexico have developed risk adjustment mechanisms. In Mexico, this new formula of resource allocation from the federal level has improved the funding situation in poorer states such as Chiapas and Oaxaca [37]. However, the impact the risk adjustment mechanism actually had on the financing situation in poorer states should not be overestimated since more funding was available in all states after the introduction of the Seguro Popular in 2003 [37]. The complexity and difficulty of balancing risks across sub-pools was also experienced in Colombia, where the indicators used did not adequately predict the population’s health needs and demand for health services in each area. [21] New and more sophisticated risk adjustment techniques are being considered [21].

### Effects of benefit package design on financial protection and access

The benefit package design including its co-payment mechanisms and rates as well as benefit ceilings for the subsidized affect the degree of financial protection and utilization rates. One important commonality across countries is that there are no co-payments for the subsidized, except in Colombia. In combination with expanded benefit packages, this could have contributed to the reduction in OOP in most countries. Likewise, this may explain increased utilisation rates noted in all countries with available data.

Another important feature are the specific and explicit lists of services covered in the benefit package. Some argue that these specific lists are potentially more effective, equitable and efficient than implicit rationing [48]. However, the comparatively larger scope of the benefit package in Costa Rica and Uruguay can be considered to have had an important impact on the relatively higher levels of financial protection. For example, in Costa Rica OOP mainly occurs when attending outpatient services such as dentist consultations or when paying for certain medicaments. In contrast, OOP on in-patient care is minimal [49], and its catastrophic health expenditure is the lowest among all the countries studied. In view of the relatively higher OOP share in Mexico, yet its relatively lower incidence of catastrophic expenditure, *Seguro Popular* seems to be able to provide financial protection for the subsidized. This suggests that even if overall OOP may not decline rapidly with high coverage because of many factors – one being that families were under-spending on health before UHC – the risk of catastrophic or impoverishing health expenditures is substantially reduced. Again, the selection of services being covered including those covered by the Catastrophic Expenses Protection Fund may explain this phenomenon.

However, except for Colombia since 2012, the scope of the benefit package in countries with separate schemes is not equal to the one for those enrolled in the contributory scheme and has resulted in lower capitation payments for the subsidized [7]. For example, average capitation payments differ quite significantly in the Dominican Republic (four times higher for contributors in 2010 [32]) and Peru [34]. Likewise, in Colombia in 2011, US$ 506 was spent per contributor compared to US$ 302 per subsidized [35]. In 2012, the government passed new regulations that aimed at all citizens having the same benefit package regardless of their health insurance coverage arrangement. Moreover, even when benefit packages are harmonized and come along with similar payment rates, effective access may still remain unequal due to service quality problems and supply side constraints that may persist especially in remote and poor areas where the subsidized live [21, 32].

### Effects of provider payment methods and purchasing arrangements on access

While all countries studied use the same provider payment methods for the subsidized and those in the formal sector schemes, with the exception of Mexico, payment rates have differed and average per capita spending for the subsidized was found to be lower than for the contributors in a number of countries. This may be due lower utilisation rates of the subsidized, or else due to hidden incentives for cream skimming, i.e. preferring some patients from one population group, which in turn can lead to the under-provision of services to financially less attractive subsidized patients. More data is needed to assess this further.

Likewise, evidence on purchasing arrangements is insufficient to determine whether the introduction of a purchaser-provider split as well as contracting of providers in all countries have increased service delivery, efficiency and equity for the subsidized. Colombia is the only country studied that manages competition among purchasers, but this has not right away resulted in more choices and competition among providers. Accordingly, Frenk (2014) suggests that countries need to further strengthen the separation of functions [17].

In sum, the lack of data on UHC progress is a limitation to assessing the effect of critical institutional design features, and there is thus need for robust and rigorous impact studies. Future research should generate more evidence on the implementation process as well as the impacts of the schemes on UHC progress, differentiated along population groups, income quintiles as well as insured versus uninsured and the contributors versus the subsidized. This is the basis to explore further options of improving coverage and also provide lessons to other countries across the globe that plan to introduce subsidization schemes.

## Conclusion

This paper explored the patterns of institutional design aspects of subsidization arrangements using state budget transfers with a focus on population groups outside formal sector employment and especially vulnerable population groups. Notably, the analysis revealed numerous commonalities but also differences across countries. A number of policy lessons can be derived from this analysis.

First of all, in all countries, the poor are fully subsidized and do not have to pay partial contributions. This is analogous to the design features that were revealed in all the other regional studies with the same research question [11–14]. The main challenge here is to ensure that the amount of transfers is sufficient and allows for sustainability of the health insurance type scheme in the long run. An important institutional design feature is the choice of the targeting method. Where a universalist approach of covering all those outside formal sector work is not (yet) feasible given the fiscal situation, a combination of both direct and indirect targeting, including socio-demographic, socio-economic and/or socio-geographic criteria, is conducive to increase enrolment rates. This design pattern is also found in Asia and Africa primarily. Equally critical is to make affiliation mandatory, which all countries except two did. Again this is similar to the design pattern found in the other regions. Other important features are the specific and explicit lists of health services covered in the benefit package and exemption of the subsidized from co-payments. One key lesson is that this has to go along with increased state budget revenues the amount of which needs to be adapted to both the (increasing) number of affiliated persons, particularly when participation is mandatory, and to the average costs of the benefit package.

With regards to pooling, countries that established integrated schemes along with mandatory coverage reduced fragmentation and have a better chance to improve equity in access and financial protection. Thus, in the medium and long term, the most desirable way for countries with separated schemes to improve equity is to merge separate schemes into one integrated health financing system without opt-out options for the better-off. In the short term, the aim could be to work towards a harmonized benefit package, at least with respect to the range of services of covered and more equal per capita spending. Yet, although a prerequisite for access, it is clear that legally granted coverage and comprehensive benefit packages on paper are not enough in view of existing quality differences between rural and urban areas and wealthy and poorer communities. There is urgent need to have sufficient funding reaching the facility, but also to allocate resources more equally within a country. Access to good quality health services can only become a reality if shortages in human resources, infrastructure and supplies are also addressed. Moreover, it is important that people are well informed about their entitlements, particularly in terms of their eligibility status for subsidization and the scope of the respective benefit package.

Despite the considerable progress achieved through the subsidization arrangements that made a difference to thousands of people, there remain concerns as to inequity in access and financial protection across income quintiles and population groups even in countries with integrated schemes. Moreover, certain population groups remain excluded as they are not eligible for subsidization, in particular near- or non-poor informal workers. There is need to expand eligibility criteria to cover the hard to reach groups as well.

In addition to favourable institutional design, expansion of population coverage to so far non-covered population groups as well as extension of the health services covered is also enhanced by economic growth that opens the necessary fiscal space for subsidization, coupled with political will. Countries with limited resources can expand coverage in a progressive way.

Overall, this analysis suggests that state budget transfers to health insurance type arrangements have been one way to expand coverage of vulnerable groups and those outside formal sector work in the studied countries. As in Africa and Asia, this financing arrangement is gaining momentum across the Latin American region and reflects the move towards a human rights based approach to health in which entitlements are no longer linked to members' direct contributions.

## Abbreviations

AUGE: Plan de acceso universal de garantias explicitas (Chile)
FONASA: Fondo nacional de la salud (Chile)
GGHE: General government health expenditure
ILO: International labour organization
ISAPREs: Instituciones de salud previsional (Chile)
MOH: Ministry of health
OECD: Organization for economic co-operation development
OOP: Out-of-pocket expenditure
PAHO: Pan America health organization
SHI: Social health insurance
THE: Total health expenditure
UHC: Universal health coverage
WHO: World health organization
